# Medical Cannabis for Gilles de la Tourette Syndrome: An Open-Label Prospective Study

**DOI:** 10.1155/2022/5141773

**Published:** 2022-03-09

**Authors:** Saar Anis, Corinne Zalomek, Amos D. Korczyn, Alina Rosenberg, Nir Giladi, Tanya Gurevich

**Affiliations:** ^1^Movement Disorder Unit, Department of Neurology, Tel-Aviv Sourasky Medical Center, Tel-Aviv, Israel; ^2^Sackler School of Medicine, Tel-Aviv University, Tel-Aviv, Israel; ^3^Movement Disorders Institute, Department of Neurology, Chaim Sheba Medical Center, Ramat Gan, Israel; ^4^School of Public Health, Epidemiology, Sackler School of Medicine, Tel-Aviv University, Tel-Aviv, Israel; ^5^Sagol School of Neuroscience, Tel-Aviv University, Tel-Aviv, Israel

## Abstract

**Objectives:**

Assessing the effectiveness and tolerability of medical cannabis (MC) treatment on Gilles de la Tourette syndrome (GTS) patients.

**Methods:**

We report on an open-label, prospective study on the effect of MC on adult GTS patients. MC mode of use was decided by the treating neurologist and the patient. *Δ*9-Tetrahydrocannabinol (*Δ*9-THC) and cannabidiol (CBD) content within MC product and monthly dose were titrated during the study. Following treatment initiation, patients were assessed after 4 and 12 weeks for efficacy, tolerability, and side effects.

**Results:**

Eighteen patients entered the study. Baseline Yale Global Tic Severity Scale- (YGTSS) Total (range 0-100) was 60.3 ± 17.1. Three patients did not reach the end of follow-up period. The most common mode of administration was smoking (80%). Following twelve weeks of treatment, a significant 38% average reduction (*p* = 0.002) of YGTSS-Total and a 20% reduction (*p* = 0.043) of Premonitory Urge for Tic Scale (PUTS) were observed. Common side effects were dry mouth (66.7%), fatigue (53.3%), and dizziness (46.7%). Three patients suffered from psychiatric side effects including worsening of obsessive compulsive disorder (stopped treatment), panic attack, and anxiety (resolved with treatment modification). Six patients (40%) reported cognitive side effects regarding time perception, visuospatial disorientation, confusion, slow processing speed, and attention.

**Conclusions:**

MC treatment demonstrates good efficacy and tolerability in adult GTS patients. Predilection for smoking rather than using oil drops requires further comparative studies to evaluate the efficacy of each. Cognitive and psychiatric side effects have to be monitored and addressed.

## 1. Introduction

Gilles de la Tourette syndrome (GTS) is a childhood-onset neuropsychiatric disorder characterized by the presence of multiple motor and one or more phonic tics that continue for at least one year [1]. GTS is a relatively common condition with a prevalence of 0.3-0.8% in school-age children [2, 3]. Tics have a pattern of waxing and waning course with a wide range of frequencies and intensities. Environmental factors such as stress, anxiety, and fatigue may transiently exacerbate tics [4]. GTS is strongly associated with many psychiatric comorbidities, including attention deficit/hyperactivity disorder (ADHD), obsessive compulsive behavior/disorder (OCB/OCD), depression, anxiety, sleep disorders, and rage attacks [1]. Current research suggests alterations to the cortico-striato-thalamo-cortical pathways, specifically the dysfunction of the dopaminergic pathway, in the pathophysiology of GTS [5, 6]. Dopaminergic activity is found to be increased in GTS. Animal trials have demonstrated that specific disinhibition of the striatum, thus altering the release of dopamine, generated motor and vocal tics demonstrative of those in GTS [7]. Due to the significant role of dopamine in the development of tics, the current Food and Drug Administration (FDA) approved pharmacotherapy for GTS has been centered around dopaminergic receptors in the synaptic cleft [8]. Therapy for GTS is tailored to the frequency and severity of symptoms. The most recent therapy guidelines have been published by the American Academy of Neurology (AAN) in 2019 [9] and the European Society for the Study of Tourette Syndrome (ESSTS) in 2021 [10–12]. Behavioral therapy, such as comprehensive behavioral intervention for tics (CBIT), is safe and effective and should be considered as an initial treatment choice. Medications for tics include antipsychotics, D_2_ antagonists, which are the most widely used drugs for the treatment of tics caused by GTS; however, these medications might cause severe side effects (SEs), such as parkinsonism, metabolic syndrome, and hyperprolactinemia, especially with chronic use, that create issues with compliance and are not sufficiently effective. Alpha-2-adrenergic agonists, vesicular monoamine transporter 2 inhibitors, benzodiazepines, antiepileptics, and botulinum toxin are also acceptable treatment approaches with varied treatment efficacy, and for refractory, “malignant” syndrome, deep brain stimulation may be advised [13, 14]. The endocannabinoid neuronal system regulates the activity of the striatal dopaminergic pathway through activation of cannabinoid receptor type 1 (CB1) located in the central nervous system, mainly in the basal ganglia [15]. Activation of CB1 receptors modulates body movement and transmission of glutamate, gamma-aminobutyric acid (GABA), norepinephrine, serotonin, and acetylcholine neurotransmitters in the brain [15, 16]. Cannabis was suggested as a possible treatment for GTS since the late 1980s in an anecdotal report by Sandyk and Awerbuch [17]. Two consecutive randomized controlled trials (RCTs), published during 2002-2003 by Mueller-Vahl et al., using tetrahydrocannabinol (THC, dronabinol), have demonstrated safety and efficacy in the treatment of tics in patients with GTS [18, 19]. After showing promising results in phase 1 [20], a recent multicenter phase 2 RCT [21] with Lu AG06466 (formerly known as ABX-1431), a modulator of endocannabinoid neurotransmission, has failed to prove effective in the suppression of tics compared to placebo. A phase 2, uncontrolled trial of THX-110, a therapeutic combination of delta 9-tetrahydracannabinol and palmitoylethanolamide, has shown improvement of tic symptoms of more than 20%, with manageable side effects [22]. An ongoing RCT called CANNA-TICS is assessing whether treatment with cannabis extract nabiximols (Sativex®) is superior to placebo in patients with chronic tic disorders [23]. In addition to these RCTs, retrospective data analysis and case series [24–27] along with multiple case reports [28–37] have been published and summarized in a recent review of the literature by Szejko et al. [38], suggesting that cannabis-based medicine (CBM) could be effective and relatively safe in suppressing tics and in the treatment of associated psychiatric comorbidities in patients with GTS. In the recent AAN guideline recommendation summary [9], cannabis-based therapies were suggested to have limited evidence to reduce tic severity, although, where regional legislation allows, physicians may consider treatment with CBM in otherwise treatment-resistant adults with clinically relevant tics (level C). Recent ESSTS guidelines state that in resistant cases, treatment with agents with limited evidence base could be considered, such as CBM [12]. This is also illustrated in the ESSTS algorithm for the treatment of tics suggesting considering alternative medication, such as CBM, in patients who do not respond appropriately to behavioral therapy and to various pharmacotherapies [11]. In Israel, although consumption of cannabis is outlawed, there is an option to allow patients to consume the drug under supervision, and at least 50,000 patients use medical cannabis (MC) regularly, particularly for pain and posttraumatic stress disorder. MC has been an approved treatment by the Ministry of Health (MOH) for resistant GTS since 2013. MC can be taken as an oil extract, through inhalation, or by smoking of dried female buds. The THC to cannabidiol (CBD) percentage within a specific cannabis product is fixed in one of the following ratios: 1 : 20, 3 : 15, 5 : 5, 5 : 10, 10 : 10, 10 : 2, 15 : 3, and 20 : 4. Varying ratios of THC : CBD percentage exert different influences. The licensed treating physician decides which ratio to recommend; however, there is insufficient data to guide which ratio to prescribe for the different severity of symptoms. In a group of 42 patients treated with MC at our GTS clinic (not part of this study cohort), we observed in a retrospective manner a significant reduction in the number and intensity of tics, as well as a decrease in premonitory urges in 83% of the patients [24]. The treatment resulted in subjective improvements in both symptom severity and quality of life, with no serious adverse events. Our preliminary results combined with publications from other centers justify the need for additional controlled studies to further evaluate and characterize the effects of MC on GTS symptoms. By using an open-label, prospective design, this study is aimed at determining the preferred method of use, efficacy, and tolerability of 12 weeks of treatment with MC in adult patients with GTS.

## 2. Materials and Methods

### 2.1. Study Design

Eighteen patients with GTS who satisfied the selection criteria, as outlined below, were chosen to participate in our open-label clinical trial based at Tel-Aviv Sourasky Movement Disorders Unit (MDU). Each subject signed a written informed consent before inclusion in the trial. In addition, since driving under the influence of cannabis is forbidden by the Israeli law, patients were instructed and gave their oral commitment to avoid driving. The study was approved by the research ethics (Helsinki) committee at our center. MC was consumed as oil extract, vaporized, or smoked dried buds. The treating neurologist (S.A.) and the patient together decided on the method of consumption during the visit before initiating treatment. The percentage of THC and CBD was preset to 10% and 2%, respectively. All patients received the same general instructions for treatment titration, which was to start with 1 drop or puff a day and increase by 1 drop or puff as needed. There was no fixed schedule for the incremental increases; thus, each patient freely raised the dose as well as the number of daily consumptions until clinical benefit was achieved or SE emerged over a follow-up period of 12 weeks. Patients were assessed 4 and 12-weeks following treatment initiation to gather data regarding treatment efficacy, tolerability, and SEs.

### 2.2. Selection Criteria

Selection criteria are as follows: (1) age 18-65 years, (2) diagnosis of GTS confirmed by the treating neurologist (S.A.) based on the DSM-V criteria [39], (3) eligibility to receive MOH MC license for GTS, (4) provided written informed consent, (5) did not regularly use cannabis in any form of self-medication prior to entering the study, (6) were not pregnant or lactating women, (7) did not have a tic disorder other than GTS, and (8) did not have concurrent physical or mental disease that could interfere with the study.

### 2.3. Assessment Procedure

Assessment of symptoms was based on clinical or telephone interviews conducted by one of the authors (S.A. or C.Z.). The primary efficacy outcome was based on the Yale Global Tic Severity Scale (YGTSS) [40]. The YGTSS is a clinician completed rating instrument and is currently the gold standard for assessing the severity of tics in children and adults. The YGTSS Tic Score (YGTSS-TS) scale quantifies the number, frequency, intensity, complexity, and interference of motor and phonic tics that occurred during the prior week. Each section is given a score from 0 to 5, for a potential total of 50. The YGTSS Impairment (YGTSS-I) scale is used to describe the impact of tics on quality of life; it is given a score from 0 to 50. Global severity score (YGTSS-Total) was calculated as the sum of the YGTSS-TS and YGTSS-I, range 0–100. Premonitory Urge for Tic Scale (PUTS) is a self-reported 9-item measurement used in the study to assess the intensity and frequency of premonitory sensory phenomena (scale of 1-4, total score 9-36) [41]. Two Likert-type scales (range 1-7, 1 = very dissatisfied, 2 = moderately dissatisfied, 3 = slightly dissatisfied, 4 = neutral, 5 = slightly satisfied, 6 = moderately satisfied, 7 = very satisfied), one for “subjective report of treatment effect on tics” and the other for “subjective report of treatment effect on general quality of life (QoL),” were used as tools to measure the subjective effectiveness of MC. Patients were additionally asked to rate the impact of MC on symptoms commonly associated with GTS, such as restlessness, tempered behavior, OCB/OCD, attention, mood, sleep, and sexual function. Lastly, participants were asked about SEs of MC treatment including psychiatric, neurological, and other somatic symptoms. During the baseline visit, data were collected on demographics, disease characteristics, previous and current pharmacological and nonpharmacological treatments, baseline YGTSS, and PUTS scales. Each patient was instructed on MC titration to implement for the following 12 weeks. Visits two and three, at 4 weeks and 12 weeks, respectively, were conducted by S.A. and C.Z. either in the clinic or by telephone. During those visits, participants were assessed using YGTSS, PUTS, and two Likert-type scales, and information about method of MC use, frequency of use, dose in grams, percentage of THC and CBD, and SE profile was collected.

### 2.4. Primary Outcomes

The primary outcomes were (1) a significant reduction in YGTSS-TS, YGTSS-I, and YGTSS-Total, (2) a significant decrease in PUTS, (3) subjective improvement of tics and QoL using a Likert-type scale, and (4) subjective percentage of improvement of tics compared to baseline. Additional aims of this study were to examine the patient's consumption habits including way of administration (smoked, inhaled, or sublingual oil extraction), dose in grams/month of MC, the percentage of THC and CBD in the formulation consumed, the frequency of use each day, and the quantity of puffs/drops per use.

### 2.5. Statistical Methods

The statistical analyses included all 18 patients who signed informed consent and completed the first baseline visit of this study. Descriptive statistics were used for demographic variables including mean, median, standard deviation (SD), and range. Friedman's two-way analysis, a nonparametric test, was used to compare the primary outcome measures between visit 1, visit 2, and visit 3 (YGTSS-TS/I/Total and PUTS change). The nonparametric Wilcoxon test was used to compare the change of MC dose, frequency, and quantity of use, as well as MC efficacy and QoL between visits 2 and 3. Significance level of nonparametric testing was 0.05. All statistics were done with SPSS version 27.

## 3. Results

### 3.1. Subjects

A total of 18 patients with a diagnosis of GTS who fulfilled the inclusion criteria were recruited at our GTS clinic between July 01, 2020, and January 01, 2021.  Table 1 gives the demographic characteristics of our patient cohort. The majority of participants were male (61%), and the median age was 30.5 and disease duration 23.5 years. Comorbidities in our cohort included a diagnosis of ADHD for 14 patients (77.8%), obsessive compulsive symptoms/disorder (OCS/OCD) for 12 patients (66.7%), history of depressive episodes for 7 patients (38.9%), and anxiety for 10 patients (55.6%). Twelve patients (66.4%) had tried pharmacological therapy for GTS (including alpha-2 agonists, dopamine blockers, benzodiazepines, and antiepileptic drugs) in the past, while only one person (5.5%) entered the study while currently being medicated for GTS (aripiprazole).

### 3.2. Premature Termination

Three of the 18 patients (16.7%) terminated the trial prematurely. The reasons for an early discontinuation were a severe depressive episode following recruitment before cannabis use in one patient, presumed lack of efficacy and dislike for the smell of cannabis (withdrew after the first visit), and worsening of obsessive thinking and compulsions in one patient (withdrew just before the second visit).

### 3.3. Primary Outcomes

**Figure 1:**
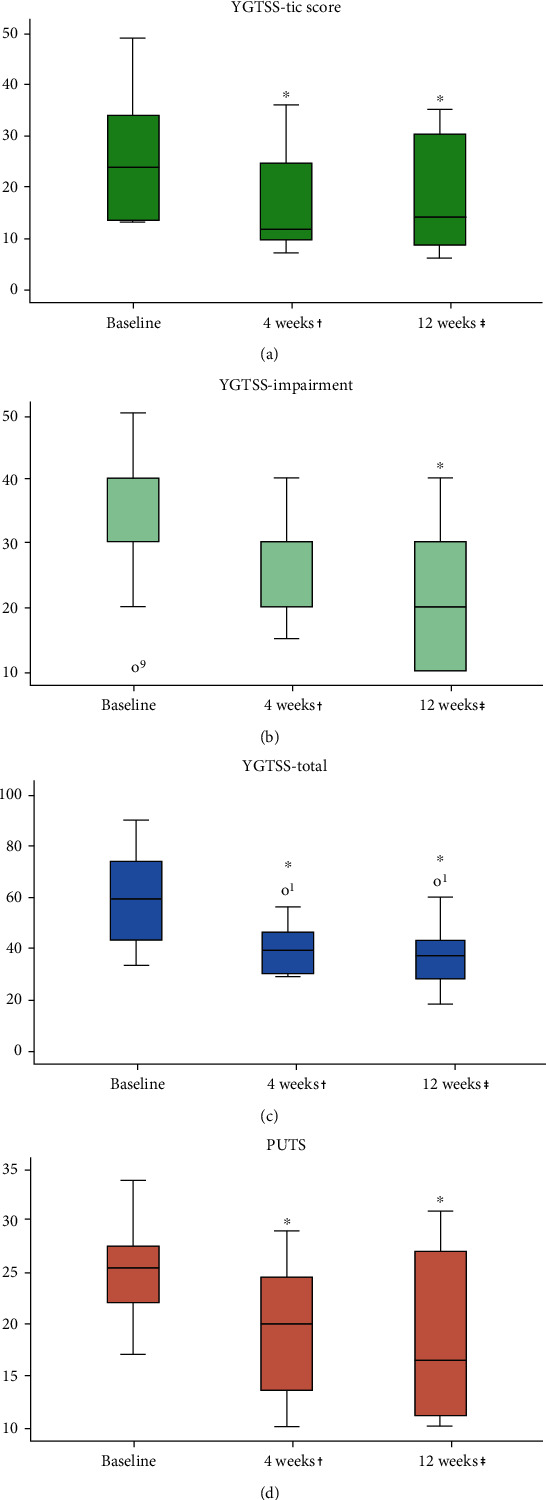
Primary outcome measures at baseline and following 4 and 12 weeks of MC use. (a) YGTSS-TS (score 0-50). (b) YGTSS-I (score 0-50). (c) YGTSS-Total (score 0-100). (d) PUTS (score 4-36). ^∗^Compared to baseline. ^†^*N* = 12, 3 subjects failed to complete midtrial evaluation, 3 subjects terminated the trial prematurely. ^‡^*N* = 15. Abbreviations: MC: medical cannabis; YGTSS-TS: Yale Global Tic Severity Scale Tic Score; TGYSS-I: Yale Global Tic Severity Scale Impairment; PUTS: Premonitory Urge for Tic Scale.

**Figure 2:**
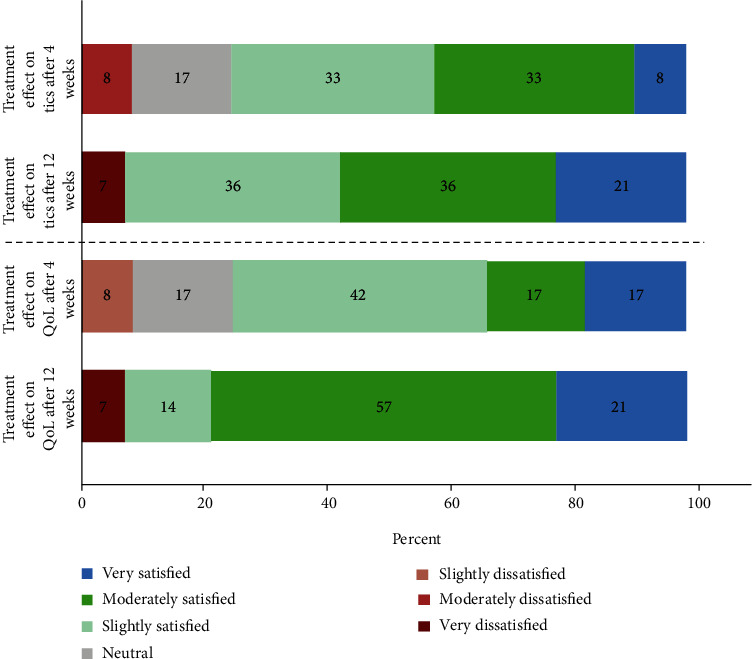
Subjective effectiveness of MC on tics and QoL using a 7-point Likert-type scale. Abbreviations: MC: medical cannabis; QoL: quality of life.

Our analysis showed a significant (*p* = 0.003) reduction in YGTSS-TS following 4 weeks of MC treatment. On average, an over 8-point reduction (25.3 at baseline to 17.0) in the YGTSS-TS was achieved, equitable to a 32.8% reduction in the motor and vocal tic score. Further decrease of YGTSS-TS to an average of 16.4 points was demonstrated following 12 weeks of MC use, signifying an average of 9-point reduction compared to baseline (*p* = 0.002), equitable to greater than 35% decrease in YGTSS-TS compared to baseline (Figure 1(a)). A nonsignificant (*p* = 0.248) average reduction of 10 points, from 35.0 to 25.0, was observed in YGTSS-I following 4 weeks of MC treatment. 12 weeks after treatment initiation, there was a further reduction in impairment due to tics, to a nadir of 40% according to YGTSS-I, statistically significant compared to baseline (*p* = 0.013, Figure 1(b)). YGTSS-Total revealed a significant (*p* = 0.001) reduction of roughly 38% in the burden of disease after 12 weeks of MC use (Figure 1(c)). A more modest but significant (*p* = 0.043) reduction was observed in PUTS from 22.9 points on average before cannabis treatment to 18.3 points on average after 12 weeks of use (Figure 1(d)). Using a Likert-type scale, we intended to subjectively estimate each patient's experience with cannabis on the influence on tics and QoL. After just 4 weeks of MC use, there was an overwhelmingly positive response demonstrated by a score of 5.1 points out of 7. Following 12 weeks, there was an even stronger indication of satisfaction, with reported scores of 5.5 and 5.7 (out of 7) for cannabis effect on tics and QoL, respectively (Figure 2). The mean subjective tic improvement following 12 weeks of MC was 53.9%. These primary results are outlined in Table 2.

### 3.4. Cannabis Consumption Characteristics

One of our goals in this open-label study was to examine our patient's consumption habits including way of administration (smoked, inhaled, or sublingual oil extraction), dose in grams/month of MC, the percentage of THC and CBD in the formulation consumed, the frequency of use each day, and the quantity of puffs/drops per use. Our results showed that the majority of patients (93%) who completed 12 weeks of cannabis use chose to consume MC through their lungs (smoking or inhalation). One patient (7%) used both sublingual oil and smoked cannabis. Another patient (7%) solely used the oil extract via sublingual administration. After 4 weeks, the average dose consumed was 16.8 grams/month. After 12 weeks, the average dose increased to 18 grams/month. All patients started on the same THC : CBD ratio (10 : 2). At the end of the study, the average ratio was 12.3 : 3.6, suggesting a higher percentage of THC was needed for our patients to achieve clinical significance. There was also a small increase in the frequency of MC per day, from 2.7 times per day after 4 weeks to 2.9 times per day on average after 12 weeks. Moreover, in proportion to more frequent, patients consumed more puffs/drops of MC with each use; at 4 weeks 7.7 puffs/drops were used on average, which rose to 10.1 puffs/drops with each use after 12 weeks. Dose, ratio, frequency, and quantity of MC used can be seen in Table 3.

### 3.5. Cannabis Influence on Comorbid Conditions and Life Habits

**Figure 3:**
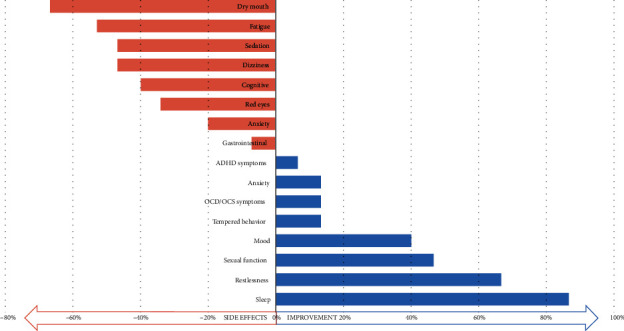
Treatment-related side effects and influence on comorbidities and life habits. Abbreviations: OCS: obsessive compulsive symptoms; OCD: obsessive compulsive disorder; ADHD: attention deficit hyperactive disorder.

We asked patients to report any benefit from MC use on comorbid conditions associated with GTS and life habits, such as sleep and sexual function. Interestingly, 40% of patients reported at least 50% improvement in mood compared to baseline. There was a significant improvement in sleep, with 9 patients reporting improved sleep preservation and 10 reporting improved sleep onset. Sexual function was improved in 47% of our patients, with reports of improved libido and two reports of improved erection. See summarized data on MC influence on comorbid conditions and life habits in Figure 3.

### 3.6. Adverse Events

The adverse events reported during the trial are shown in Figure 3. The most common adverse event was dry mouth (67%), followed by fatigue (53%), and sedation and dizziness (47%). A substantial number (40%) reported cognitive SEs including altered time perception (*n* = 1), visuospatial orientation (*n* = 1), attention (*n* = 1), confusion (*n* = 2), and speed of processing (*n* = 1). Three patients suffered from anxiety secondary to cannabis use, and one additionally suffered from a panic attack soon after initiation of treatment. The panic attack was due to titration practices that differed from what was instructed with the use of a high dose (more than 10 puffs) soon after initiation. With a substantial reduction of THC content (from 10% to 3%) of his MC, he had no panic attacks, but he felt little improvement of tics. Of note, after study termination he continued follow-up, and through slow titration and gradual increase in THC percentage to 10%, he was able to achieve a better response with no SEs. One patient had to stop the study due to worsening of his OCD, as mentioned above.

## 4. Discussion

The results of this pilot open-label prospective study are in line with our clinical impression that MC may be an effective treatment for patients with GTS. The main outcome measures YGTSS-TS, YGTSS-I, and YGTSS-Total showed a significant improvement of 35, 40, and 38 percent, respectively, after 12 weeks of use. PUTS showed significant improvement as well with a modest decrease of 20% after 12 weeks of MC. Using Likert-type scales [1–7], patients self-reported significant satisfaction with cannabis with tics and QoL with scores of 5.5 and 5.7, respectively. To illustrate the results clinically, according to YGTSS scale, a typical patient with moderate-marked severity of tics and impairment successfully improved to mild-moderate severity through a course of treatment with MC. Several previous reports of GTS improvement with the use of MC have been published. Two small placebo-controlled studies with 36 participants [18, 19] suggested that THC capsules may significantly improve tic severity in patients with GTS compared with placebo. Analysis of placebo effect in those studies showed no average reduction in clinical global improvement (CGI), YGTSS, and premonitory urge in the 6-week randomized trial [18], and in the single-dose crossover trial, 25% of the placebo group showed mild improvement (average of 7% global improvement) compared to 83% of patients treated with THC capsules (average of 35% global improvement) [19], manifesting a very mild placebo effect. A more recent publication from the same group [25] found that cannabis-based treatment (for medical cannabis, THC : CBD ratio was not published) resulted in a subjective improvement of tics of about 60% in 85% of treated cases. In this study, our patients collaborated with the treating neurologist to select the method of administration of MC that best fits personal preferences. Interestingly, patients had a predilection for cigarette smoking/inhaled buds via a designated vaporizer (93%) over the extracted oil. This could be explained by the pharmacokinetics of THC. THC when inhaled reaches the peak plasma concentration in 8-12 minutes, while sublingual oil takes longer to absorb with the first measurable plasma levels at 30 minutes and peak concentration at 3-4 hours [42, 43]. Additionally, smoking THC achieves a higher plasma concentration (*C*(max), (microgram/L)^6^) compared to sublingual oil [43, 44]. Collectively, this may show a benefit in prescribing inhaled cannabis, specifically, to reduce the symptoms of GTS. It is important to evaluate the increase in THC percentage and MC total monthly dose during the trial. During the study, patients were able to request for a change in THC percentage if they did not feel the maximum effects of cannabis with their current prescription. After 4 weeks, patients on average used 16.8 g/month of 10 : 2 THC : CBD, which corresponded to 8.9 mg THC/day. After 12 weeks, patients showed a preference for a higher THC percentage (12.33%) compared to baseline (the baseline default was 10%, which is the recommended starting point in the Israeli guidelines for using MC for GTS) and an increase in dose to 18 grams/month, which corresponded to 12 mg THC/day. In a cohort of 25 GTS patients with chronic MC use (average time of use 4.8 ± 3.0 years) in our MDU, the average THC percentage is 15.5 ± 4.6 and monthly cannabis use is 33.7 g/month (~1.1 g/day), corresponding to 27 mg THC/day. Both sets of data support the findings of other studies that showed “THC-rich” MC strains to be more effective and better tolerated compared to nabiximols (containing 2.7 mg THC and 2.5 mg CBD per puff) and dronabinol (oral synthetic form of THC) [25, 37]. This suggests that a higher THC percentage and total daily dose may work better in GTS. We hypothesize that if further followed, our patients would increase their daily and monthly cannabis dose and achieve further improvement in their symptoms. The influence of MC on comorbid conditions was less impressive in our cohort. Symptoms of ADHD were reported to improve in only one patient and OCS/OCD in two patients. That contrasts with a recent report suggesting a 53% improvement for ADHD and 38% for OCS/OCD [25]. However, as previously mentioned, several other comorbid conditions did substantially improve. Forty percent of our patients reported improved mood, and 72% reported improvement in sleep. In the available literature, there are contradicting results about the influence of cannabis on sexual function, with some articles indicating improvement [45, 46] and others highlighting hazardous effects like erectile dysfunction in men [47–49]. In our study, 39% of patients reported that cannabis contributed to improvement in sexual function (desire/libido, erection), which may be in part due to mood improvement. The most common SEs reported by our patients were neurological. About 50% reported dizziness and sedation, and 40% reported cognitive SEs such as altered time perception, visuospatial orientation, confusion, and speed of processing; all have been previously described before [50, 51]. Our results show higher rates of neurological SEs compared to those reported by Milosev et.al [25]. In contrast to our high rate of reported cognitive SEs, the study done by Muller-Vahl et al. found that treatment up to 10 mg delta(9)-THC over a 6-week period has neither acute nor long-term cognitive deficits [18]. This discrepancy in SEs with MC use may be due to the subjective components of our data collection and patient autonomy in the titration process. It is important to note that while 2 patients (11%) reported improvement in anxiety following treatment, 3 patients (17%) suffered from anxiety following the initiation of cannabis, one patient terminated his participation in the study, and one patient suffered from panic attacks which resolved after a substantial decrease in THC percentage. The role of CBM in the treatment of anxiety is an ongoing debate with an increasing amount of literature investigating its impact [52]. A large meta-analysis of more than 100,000 people from the general population suggests a positive association between anxiety disorders and cannabis use [53], while another large review proposes that cannabis may be effective in alleviating anxiety, although current evidence is equivocal [52]. In a recent review published in JAMA Psychiatry, there was found to be an increase in the risk of developing depression with adolescent use of cannabis, but no associations were mentioned about anxiety [54]. Our study has several limitations: (1) this is an open uncontrolled study based on clinical and telephone interviews; however, since all patients were investigated by an author experienced in GTS (S.A. and C.Z.), we believe the data to be reliable; (2) the sample size is still relatively small, yet all primary outcome measures reached statistical significance; (3) diagnoses of comorbidities were made only based on a semistructured clinical interview; (4) an effective dose of cannabis is difficult to quantify because of the different contents of cannabinoids and inhalation patterns; the bioavailability of THC following inhalation ranges between 2 and 56%, varying based on the subject's length of inhalation, lung capacity, and temperature of cannabis heating [55]; (5) the follow-up period of this study was relatively short. When comparing to our chronic MC users, we suggest that higher doses of MC per day and stronger strands (with more THC percentage) could provide added benefit.

## 5. Conclusions

Our results are in line with a number of other studies suggesting that MC is effective and well tolerated in adults with GTS. From our data, it is suggested that MC might be a treatment option for resistant TS patients, and MC has a significant effect on tics, premonitory urges, and patients' overall quality of life. In our sample, patients favored THC-rich cannabis strands and smoking/inhaling MC over sublingual oil. One has to be aware of the neurological and cognitive SEs of MC and monitor them during treatment. The limitations of our study due to the open, uncontrolled design emphasize the necessity for controlled studies to further evaluate the role of MC in this disorder.

## Figures and Tables

**Table 1:** Demographic characteristics of study participants.

Characteristics	Cohort group (*N* = 18)
Sex	
Male	11
Female	7
Age (years)	
Median	30.5
Range	20-50
Disease duration (years)	
Median	23.5
Range	8-44
Level of education (years)	
Median	12.5
Range	8-17
Employment	
Unemployed	6
Marital status	
Married	9
Single	9
Family history of tics	7
Diagnosis of ADHD	14
OCS	12
History of depressive episode/s	7
Anxiety	10

Abbreviations: OCS: obsessive compulsive symptoms; ADHD: attention deficit hyperactive disorder.

**Table 2:** Primary outcome efficacy measures (baseline and week 4 and 12 weeks after treatment initiation) for MC treatment.

Rating scale	Baseline		4 weeks^†^			12 weeks^‡^		
	Mean	SD	Mean	SD	Baseline to 4-week change *p* value (Friedman two-way analysis)	Mean	SD	Baseline to 12-week change *p* value (Friedman two-way analysis)
YGTSS-TS	25.3	10.5	17.0	10.7	0.003^∗^	16.4	10.1	0.002^∗^
YGTSS-I	35.0	11.0	25.0	7.9	0.248	21.0	10.0	0.013^∗^
YGTSS-Total	60.3	17.1	42.0	13.3	0.032^∗^	37.4	14.6	0.001^∗^
PUTS	22.9	5.5	19.4	6.7	0.043^∗^	18.3	7.4	0.043^∗^
Subjective percentage of tic improvement following treatment	—	—	50.0	23.8	—	53.9	28.8	—

^†^ *N* = 12, 3 subjects failed to complete midtrial evaluation, 3 subjects terminated the trial prematurely. ^‡^*N* = 15; ^∗^statistically significant. Abbreviations: MC: medical cannabis; YGTSS-TS: Yale Global Tic Severity Scale Tic Score; TGYSS-I: Yale Global Tic Severity Scale Impairment; PUTS: Premonitory Urge for Tic Scale; SD: standard deviation.

**Table 3:** MC consumption characteristics during trial.

	Baseline		4 weeks^†^		12 weeks^‡^	
	Mean	SD	Mean	SD	Mean	SD
MC average monthly dose (grams)	—	—	16.8	5.4	18.0	5.6
THC/CBD %	—	—	10/2		12.3/3.6	5.3/3.5
MC use times per day	—	—	2.7	0.8	2.9	1.4
Quantity in each use (puffs/drops)	—	—	7.8	5.4	10.1	4.7

^†^ *N* = 12, 3 subjects failed to complete midtrial evaluation, 3 subjects terminated the trial prematurely. ^‡^*N* = 15. Abbreviations: MC: medical cannabis; THC: tetrahydrocannabinol; CBD: cannabidiol; SD: standard deviation.

## Data Availability

The data that support the findings of this study are available from the corresponding author upon reasonable request.
